# Polyunsaturated Fatty Acids and Their Immunomodulatory Actions in Periodontal Disease

**DOI:** 10.3390/nu15040821

**Published:** 2023-02-05

**Authors:** Jeneen Panezai, Thomas van Dyke

**Affiliations:** 1 Department of Applied Oral Sciences, The Forsyth Institute, Cambridge, MA 02142, USA; 2 Department of Microbiology, Faculty of Life Sciences and Informatics, Balochistan University of Information Technology, Engineering and Management Sciences, Quetta 87300, Pakistan; 3 Centre for Clinical and Translational Research, The Forsyth Institute, Cambridge, MA 02142, USA; 4 Department of Oral Medicine, Infection and Immunity, Harvard Faculty of Medicine, Boston, MA 02115, USA

**Keywords:** fatty acids, periodontitis, inflammation

## Abstract

Polyunsaturated fatty acids (PUFAs) are a diverse set of molecules with remarkable contributions to human physiology. They not only serve as sources of fuel but also cellular structural components as well as substrates that provide bioactive metabolites. A growing body of evidence demonstrates their role in inflammation. Inflammation in the presence of a polymicrobial biofilm contributes to the pathology of periodontitis. The role PUFAs in modulating immuno-inflammatory reactions in periodontitis is only beginning to be uncovered as research continues to unravel their far-reaching immunologic implications.

## 1. Introduction

Periodontitis is a chronic disorder characterized by the inflammatory breakdown of tooth supporting tissues including periodontal ligament and alveolar bone, subsequently resulting in tooth loss. It is well understood from both human and animal studies that the periodontal tissue degradation occurs due to an immunoinflammatory response. Such a response has been associated with dysbiosis of the subgingival microbiota which complicates it further [1]. Additionally, the host response will be determined by the individual’s immunotype and their immune fitness towards a biofilm.

Accumulating evidence shows that resolution of inflammation is a tightly orchestrated process which involves specific pro-resolving mediator pathways and fatty acid derived specialized pro-resolving mediators or SPMs [2]. The key events of resolving inflammation include eliminating inflammatory cells and re-establishing tissue homeostasis. Impairment in any of the components responsible for resolving acute inflammation will allow its progress to a persistent inflammatory state which, as we know, is the underlying cause for many non-communicable diseases [3]. The complex chronicity of periodontitis may be a manifestation of deficient resolution pathways. The efficacy and success of a resolution response depends upon the quantitative expression of SPM receptors, enzymatic synthesis, intracellular signaling and, most importantly, bioavailability that depends on a diet rich in essential polyunsaturated fatty acids. In this review, we highlight the biochemical, metabolic, immunologic and inflammatory aspects of *n*−6 and *n*−3 polyunsaturated fatty acids (PUFA) and their immunomodulatory actions in periodontitis.

## 2. Fatty Acids

Fatty acids (FAs) are long-chain carboxylic acids. They are the residues that form lipids. The basic structure of a fatty acid residue comprises straight acyl chains with a carboxylic acid group at one end and a methyl group at the other end. FAs can be saturated which contain no double bonds, monounsaturated containing a single double bond and polyunsaturated containing two or more double bonds. Their double bond representation determines the biologic properties of the lipids; long and saturated FAs are found in fats that are solid at room temperature, whereas shorter and more unsaturated FAs form lipids that are liquid at room temperature (referred to as oils). The order of numbering carbon atoms in FAs starts with the carbon in the carboxyl group (COOH) which is designated as C1 and the carbon atom that is furthest from the carboxyl group is denoted by the letter omega–ω or *n*. PUFAs comprise of four families classified according to the ω or *n* carbon. They include the *n*−3 series derived from α-linolenic acid (ALA,18:3, ω−3); the *n*−6 Series derived from cis-linoleic acid (LA,18:2, ω−6); the *n*−9 series derived from oleic acid (OA, 18:1, ω−9) and the *n*−7 series derived from palmitoleic acid (PA, 16:1, ω−7). The affiliation of an FA to a series of *n*−3, *n*−6 or *n*−9 fatty acids is determined by the distance from carbon ω (or *n*) to the first double bond between carbon atoms in the hydrocarbon chain (-C = C-). Thus, the chemical structure of FAs is interpreted by the number of carbon atoms, the number of double bonds and the group ω [4].

### 2.1. Metabolism

**Figure 1:**
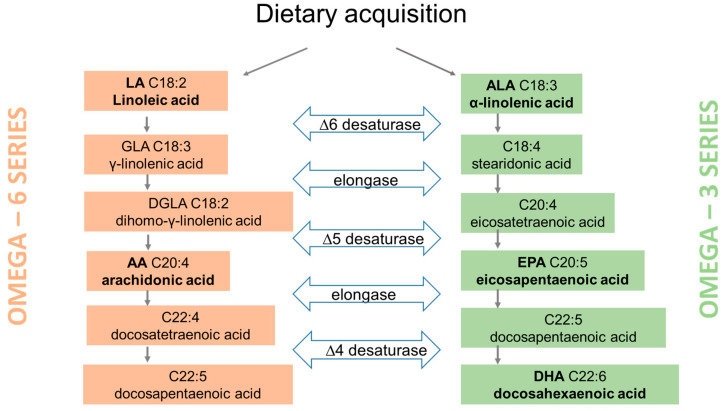
Biosynthetic pathways for omega 6 and omega 3 polyunsaturated fatty acids. Those highlighted in bold are known for their immunomodulatory actions in inflammation.

PUFAs originate from primary producers (photosynthetic marine and freshwater microalgae and bacteria) in food webs and animals can only modify them by bioconversion and elongation [5]. Humans do not possess enzymes capable of forming double bonds in fatty acid chains and are therefore unable to produce LA and ALA in the sufficient amounts. Both LA and ALA give rise to essential unsaturated fatty acids of high physiological significance and therefore must be acquired through dietary sources (Figure 1). Once consumed through diet, more than 90% of FAs are absorbed into cells via FA transporters [6]. Once inside the cell, they are converted to FA acyl-CoA thioesters, which are substrates for three metabolic pathways: beta oxidation pathway for ATP production; synthesis of triglycerides, cholesterol esters and polar lipids (phospholipids and sphingolipids) and elongation/desaturation reactions generating long chain PUFAs from the initial C18 precursors. The latter conversion occurs in the endoplasmic reticulum through consecutive elongation and desaturation reactions synthesizing longer-chain PUFAs as shown in Figure 1 [7]. The substrates for the synthesis of longer PUFAs, LA and ALA, compete for the same enzymes (elongase and desaturase) and yield arachidonic acid (AA), eicosapentaenoic acid (EPA), and docosahexaenoic acid (DHA). The incorporation of PUFAs in cell membranes contributes to their fluidity, which plays an important role in determining correct hormone-receptor binding [8].

Interestingly, there are differences found between men and women in their capacity to generate long-chain PUFAs. In young women, this capacity is enhanced generating more long chain PUFAs from ALA, which may be due to the effects of estrogen [9].

### 2.2. Bioactive Metabolites: Eicosanoids and SPMs

**Figure 2:**
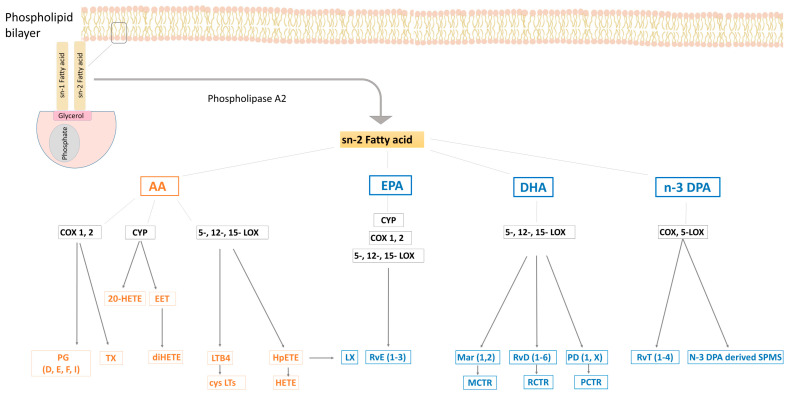
Metabolic pathways for n-6 (orange) and n-3 (blue) polyunsaturated fatty acids. COX, cyclooxygenase; CYP, cytochrome P450; CysLTs, cysteinyl leukotrienes; DHA, docosahexaenoic acid; DPA, docosapentaenoic acid; EET, epoxyeicosatetraenoic acid; EPA, eicosapentaenoic acid; HETE, hydroxy eicosatetraenoic acid; HpETE, hydroperoxy eicosatetraenoic acid; LTB4, leukotriene B4; LOX, lipoxygenase; LX, lipoxin; MaR, maresin; MCTR1, maresin conjugates in tissue regeneration 1; PCTR, protectin conjugates in tissue regeneration; PD, protectins; PG, prostaglandin; RvD, D-series resolvin; RvE, E-series resolvin; RvT, thirteen-series resolvin; Tx, thromboxane.

Eicosanoids are a family of fatty acid metabolites derived from the 20-carbon PUFAs such as EPA, DGLA and AA. The eicosanoids from AA are derived from its hydrolysis in membrane glycerophospholipids by cytosolic phospholipase A_2_ as shown in Figure 2 [10]. Since they are not stored, eicosanoids are promptly synthesized de novo after cell activation through a ligand-receptor interaction that stimulates the expression of phospholipase A_2_, cleaving AA from membrane phospholipids, where AA is found in high abundance [11]. The liberated AA will be oxygenated by three oxygenases: cyclooxygenases (COXs), P450 cytochrome epoxygenases (CYP450), and lipoxygenases (LOXs) [12].

Mammalian tissues have a wide distribution of COXs where it is expressed in two isoforms, COX−1 and COX−2 [13]. The activation of COX leads to the synthesis of prostaglandins (PGs) and thromboxanes (Txs), mediators that are collectively known as prostanoids. The oxygenation of AA will generate series 2 PGs (PGD_2_, PGE_2_, PGI_2_, and TxA_2_), whereas series 1 PGs (PGD_1_, PGE_1_, and TxA_1_) and series 3 PGs (PGD_3_, PGE_3_, PGI3, and TxA_3_,) are oxygenation products of DGLA and EPA, respectively [14]. The role of aspirin in the biosynthetic pathway of eicosanoids is vital as irreversibly acetylates COX−2 enzyme which then oxygenates AA to form 15(R)- hydroxyeicosatetraenoic acid (15(R)-HETE) and EPA to form 18(R)-hydroxyeicosapentaenoic acid (18(R)-HEPE). Both 15(R)-HETE and 18 (R)-HEPE are precursors to some of the SPMs [15].

Lipoxygenases (LOX) are nonheme iron-containing enzymes that are categorized according to their oxygenation of specific positions in AA: 5-LOX, 12-LOX, and 15-LOX [16]. The 5-LOX enzyme is well known for its ability to generate leukotrienes (LTs). It oxygenates AA to 5- 5(S)- HpETE, which is further converted to the unstable leukotriene A_4_ (LTA_4_), which is either converted to leukotriene B_4_ (LTB_4_) or leukotriene C_4_ (LTC_4_) in platelets and endothelial cells. LTC_4_ is further degraded by peptidases to form LTD_4_. Collectively, LTC4, LT_D_4, and LT_E_4 are named cysteinyl LTs (cysLTs) and are known to be produced in leukocytes only [17]. The 15-LOX enzyme oxygenates AA to 15-HpETE, which is the precursor of lipoxins (LX) A_4_ and B_4_ and belong to SPMs due to their pro-resolving characteristics (detailed below) [18].

CYP450s are a family of heme-containing monooxygenases that can metabolize AA into epoxyeicosatrienoic acids (EETs) (). Upon hydration with soluble epoxide hydrolase (sEH), EETs undergo a rapid conversion to dihydroxyeicosatrienoic acids (DHETs) which are more stable and less biologically active [19].

SPMs were identified after being isolated from inflammatory exudates. They are potent bioactive lipid mediators derived from AA, EPA, DPA and DHA [18]. These omega−3 FAs are metabolized by the same COX-, LOX and CYP-pathways generating resolvins (Rv), protectins (PD), maresins (MaR) and lipoxins (LXs). SPMs orchestrate the events involved in the resolution of acute inflammation by reducing further leukocytic infiltration, stimulating efferocytosis and exerting anti-inflammatory actions that promote healing [20]. SPMs mediate their pro-resolution actions through cell-surface G-protein coupled receptor (GPCRs). SPMs can activate more than one specific GPCR in a stereospecific manner generating downstream signals, which are transduced into pro-resolving functions. These are discussed in detail in our previous publication [3].

## 3. PUFAs in Immunity and Inflammation

### 3.1. N−6 Fatty Acids

The *n*−6 FAs are structural components of membranes and determine membrane fluidity, signal transduction as well as the expression of cellular receptors. Their biochemical function as precursors of eicosanoids is crucial as eicosanoids are considered to be locally acting hormones that are involved in the modulation of renal and pulmonary functions, vascular tone and inflammation. The cytochrome P−450 metabolites (EETs, DiHETEs and HETEs) are important paracrine factors and second messengers with regulatory functions in pulmonary, cardiac, renal, and vascular systems as well as modulating inflammatory and growth responses, whereas LXA_4_ and LXB_4_ are potent anti-inflammatory mediators [21]. Studies have shown that increasing dietary intake of *n*−6 FAs results not only in increased incorporation of AA into inflammatory cells, but also the production of inflammatory eicosanoids [22,23].

A diet comprised of high *n*−6 FAs and low *n*−3 FAs i.e., a higher *n*−6/3 ratio, appears to lower immune cell function [24]. This effect is undesirable in many ways as long-term effects can result in lower immunity. Currently, the *n*−6/3 ratio in a typical Western diet is 20-fold higher than what it was hundred years ago [25]. As we know, a high *n*−6 FA diet leads to increased incorporation of AA in immune cell membranes. In neutrophils, monocytes and lymphocytes, almost 20% of the membranous FAs are AA as opposed to just 1% EPA and 2.5% DHA [26]. The high AA content ensures an increased supply of its metabolites; the pro-inflammatory eicosanoids, which can predispose our bodies to supra-physiologic inflammatory responses and eventually perpetuate low-grade inflammation [27]. However, *n*−6 FA does remain an essential requirement for the growth and maintenance of immune cells and tissues. An abundance of in vitro evidence exists for the role of AA metabolites and their regulatory role in immune cell development and functions, including monocyte growth and differentiation, Th1 and Th2 cytokine regulation, T cell proliferation and migration, antigen-presenting cell functions and macrophage TNF-α and IL−1regulation [28,29,30,31,32,33]. Also, lymphocytes preferentially incorporate *n*−6 fatty acids during growth and proliferation *in vitro*. This can be explained by the fact that the mounting of an immune response requires increased cell proliferation in the lymph nodes, which in turn would demand an increased amount of PUFA. [34]. AA derived prostanoids, especially PGE_2_, influence T cell activation depending on its concentration. At low concentrations, it inhibits T cell activation and differentiation, whereas at high concentrations, PGE_2_ enhances T cell proliferation [35]. PGD_2_ also exerts different effects but these are not concentration-dependent; rather receptor (type) dependent. PGD_2_ engages with both DP1 and DP2 receptors. Engaging with DP1 promotes T cell apoptosis while DP2 delays Th2 apoptosis [36]. Studies examining the role of TXA_2_ in human T lymphocytes revealed an inhibitory effect on T cell proliferation and cytokine production [37]. Leukotrienes LTD_4_ and LTE_4_ on the other hand are known to enhance Th2 cell activation and cytokine production. This effect is further amplified in the presence of fellow eicosanoid PGD_2_ [38]. The AA derived pro-resolving lipoxins play an important role in T-cell mediated inflammation as well. Aspirin-triggered LXA_4_ and LXB_4_ inhibit production of TNFα in anti-CD3 antibody stimulated T lymphocytes [39].

Based on several lines of evidence, *n*−6 FAs are considered pro-inflammatory. These include the membrane AA and its oxygenated products, the association of plasma *n*−6 FA levels with certain inflammatory diseases and augmented autoimmunity in certain diseases [40]. Non-metabolized AA alone is capable of exerting direct effects on cell membranes as seen in its involvement in the production of reactive oxygen species (ROS), partly via NADPH oxidase NOX−2 which is located in the plasma membrane [41,42]. Non-metabolized AA can also alter the mechanical properties of the bilayer, thereby modulating the function of membrane channels and perturbing the localization of transmembrane receptors [43,44].

Paradoxically, the *n*−6 FAs have demonstrated protective effects in immune-mediated inflammatory diseases. An interesting finding has highlighted AA’s role in preventing pro-inflammatory signaling cascades indirectly [44]. Zhang et al. discovered that AA not only prevented the TLR4 complex formation with accessory proteins which is induced by saturated fatty acid but also the induction of pro-inflammatory cytokines in cultured cardiomyocytes and macrophages. This was due to AA’s ability to directly bind to TLR4 co-receptor, myeloid differentiation factor 2 (MD2) which prevented saturated fatty acids from activating TLR4 pro-inflammatory signaling pathway [44].

The anti-inflammatory effects *n*−6 FAs are similar to *n*−3 FAs and have been observed in other studies where *n*−6 FAs induced the production of nuclear transcription factors, enzymes, and cytokines in human cells [45]. Similar to the effects of DHA and EPA, GLA enhanced levels of the transcription factor peroxisome proliferator-activated receptor-gamma (PPAR-γ), which propagates anti-inflammatory effects decreased production of pro-inflammatory cytokines including interleukins (IL) 6 and 8 [45].

### 3.2. N−3 Fatty Acids and SPMs

Increased consumption of *n*−3 FAs, including EPA and DHA, results in increased proportions of those fatty acids in inflammatory cell membranes [46,47]. The incorporation of EPA and DHA into inflammatory cell membranes occurs in a dose dependent manner whilst outcompeting AA. As a result, less substrate AA becomes available for the synthesis of inflammatory eicosanoids by inflammatory cells decreasing their production of PGE_2_, thromboxane B_2_, LTB_4_, and LTE_4_ [48]. With increased availability of EPA and DHA in membranes, the inflammatory eicosanoids not only decrease, but an alternate family of mediators are produced including EPA derived eicosanoids (PGE_3_, LTB_5_), endocannabinoids, and SPMs (E-series and D-series resolvins, protectins and maresins). EPA derived eicosanoids are less biologically active than those produced from AA [49,50]. Being structurally different, the eicosanoid receptors have a lower affinity for the EPA-derived mediators [51].

With increased dietary intake of DHA, an increase in the activity of phagocytes (neutrophils and monocytes) occurs. An intake of a DHA rich fish oil (3 g per day) containing 54% DHA can increase the phagocytic activity of neutrophils and monocytes by 62% and 145% respectively [52]. These changes were not observed with EPA rich fish oil [53]. This impact on phagocytes shows DHA’s immunomodulatory strength in an acute inflammatory response. Nuclear factor kappa B (NFκB) is an important transcription factor involved in inflammatory responses. It is the main transcription factor required for up-regulating the genes encoding inflammatory cytokines, adhesion molecules as well as COX−2 [54]. When activated by extracellular inflammatory stimuli, NFκB’s inhibitory subunit (IκB) undergoes phosphorylation, which then allows translocation of the remaining NFκB dimer to the nucleus [55]. Both EPA and DHA can reduce NFκB activation in response to endotoxin in cultured macrophages and human monocytes due to decreased IκB phosphorylation [56,57].

The modulatory actions of *n*−3 FAs on T cells are generally suppressive in nature and specific cell responses are modulated according to the T cell subtype [58]. These suppressive actions are thought to be mediated through the perturbation of lipid rafts in the plasma membrane [59]. Lipid rafts can be defined as dynamic nanoscale domains formed via lipid-lipid and lipid-protein interactions. Incorporation of *n*−3 FAs in T helper cell membranes destabilizes the rafts resulting in the displacement of many signaling proteins necessary for T cell activation, including the Src family kinases Lck and Fyn [60,61,62]. Both EPA and DHA affect the motility of T cells as their membranous incorporation interferes with cytoskeletal rearrangements [63]. Furthermore, *n*−3 FAs increase the formation of M2 macrophages, also known as pro-resolving or regulatory macrophages, which then induce the differentiation of T cells into regulatory T cells [64].

SPMs are potent anti-inflammatory mediators which were discovered as distinct EPA- and DHA- derived mediators. They share some of the basic pro-resolving and protective actions of lipoxins with great potency in several inflammatory disease models. Distinct SPM facilitate the resolution of inflammation and accelerate tissue regeneration and tissue repair [65]. SPMs suppress the synthesis of pro-inflammatory cytokines including IL−1, IL−6, and IL−8 via down-regulation of the NFκB pathway [15]. This, in addition to halting leukocyte infiltration into inflamed tissues, distinguishes the EPA-derived resolvins (E-series resolvins), DHA-derived resolvins (D-series resolvins), and DHA-derived protectins as immunoresolving agents [15]. Maresins, also derived from DHA, stimulate phagocytosis whilst reducing neutrophil infiltration [66]. 13(S),14(S)-epoxymaresin also inhibits the production of LTB4 derived from AA through direct inactivation of the LTA_4_ hydrolase enzyme, which catalyzes the conversion of leukotriene A4 into the pro-inflammatory metabolite, LTB4 [67]. SPMs also promote the the return to a homeostatic milieu by removing apoptotic cellular debris from tissues and limiting the formation of free radicals [68]. The bioactions of SPMs occur within a low nanomolar range as demonstrated by in vitro and in vivo studies [69].

## 4. Immunomodulatory Impact in Periodontitis

### 4.1. Periodontitis

Periodontitis is a highly prevalent oral inflammatory disease in adult populations with rates ranging from 30–50% in the United States and 7% of the population globally [70,71]. Severe periodontitis is the main cause of tooth loss in adults, which is preceded by the mobility and drifting of teeth [72]. The risk determinants (non-modifiable risk factors) for periodontitis include age, gender, ethnicity, and genetics while smoking, diabetes mellitus, obesity, socioeconomic status and inflammophilic periodontal bacteria are modifiable factors [73,74].

The classification of periodontal diseases comprises staging and grading. The four stages of periodontitis depend on the severity of disease as well as the complexity of its management, while the grading of the disease is based on the rate of its progression (grade A: slow rate of progression, grade B: moderate rate of progression, grade C: rapid rate of progression) [75].

In health, protection against periodontitis is determined by the immune fitness of the host and how it combats the microbial challenge in periodontal tissues to allow a return to homeostasis [76]. However, if the microbial challenge and inflammatory tissue destruction persist due to any underlying dysfunction in the host’s immunity, the lesion can progress to a state of chronic inflammation. Alongside environmental factors, immune fitness is also determined genetically. Suspicions regarding single nucleotide polymorphisms (SNPs) in cytokine genes increasing the host’s susceptibility have led to numerous studies exploring the association. The pro-inflammatory IL−1 gene cluster polymorphisms have been shown to be associated with periodontitis [77]. Similarly, polymorphisms in the IL−8 and IL−4 genes have been shown to influence susceptibility of periodontitis. A recent study demonstrated that haplotypes formed by three SNPs in the IL−8 gene were associated with periodontitis susceptibility [78]. These genetic variants also seem to influence the periodontopathogenic colonies which further complicate the disease. For example, IL−6 haplotypes (polymorphisms rs 2069827 and rs 2069825) were shown to be associated with *Aggregatibacter actinomycetemcomitans* counts in subgingival plaque samples [79].

In the case of IL−4, an anti-inflammatory cytokine, two haplotypes in its gene conferred different extents of susceptibility. In individuals carrying the genotype TCI/CCI, susceptibility towards periodontitis was five times higher whereas those carrying genotype CTI/TTD appeared to have low susceptibility or better protection from developing periodontitis [80].

Conventional periodontal therapy is aimed at reducing or eliminating oral bacterial biofilm via mechanical debridement and/ or chemical plaque control, often supplemented with antibiotics. In addition, modifiable risk factors including smoking cessation and glycemic control have been addressed to improve periodontal parameters. Conventional therapy often requires periodontal maintenance due to bacterial recolonization of the subgingival environment following treatment [81]. Cortellini and colleagues reported a higher rate of recurrence in patients treated with surgical flap treatment after 20 years of follow-up [82]. This shows that periodontal health is largely associated with one’s immune fitness. Therefore, the emergence of host modulation as an additional therapeutic approach in the treatment of periodontitis is attractive. Most importantly, host modulation therapy aims to address the chronic insufficiency of resolution of inflammation, which in turn would minimize tissue destruction and enhance tissue restoration in the periodontium by downregulating destructive pro-inflammatory mechanisms and upregulating protective and/or regenerative components of the host response [83].

### 4.2. Anti-Inflammatory Actions

The overall positive effect of *n*−3 FA supplementation in the treatment of periodontitis has been observed via significant reduction of pocket depth and clinical attachment levels [84]. By decreasing the host inflammatory response, *n*−3 FAs prevent tissue breakdown, which reduces the availability of protein-derived energy source for periodontopathogens (). By decreasing IL−1β and TNF-α levels, *n*−3 FAs have a stabilizing effect on collagen fibers, as well as a modulating effect on the inflammatory destruction of gingival connective tissue [85]. Similar tissue regenerative actions were observed by Hankenson and colleagues in medial collateral ligament (MCL) fibroblasts where their exposure to EPA enhanced overall collagen synthesis and the proportion of collagen produced. In addition to IL−1β and TNF-α, EPA also decreases PGE_2_ production [86]. The decrease in pro-inflammatory cytokines is in fact a result of decreased AA: EPA ratio in the membrane phospholipids of mononuclear cells [87]. Host modulation by dietary *n*−3 FAs in periodontal soft tissue was observed three decades ago when Alam and co-workers showed how dietary *n*−3 FAs decreased levels of AA by half and PGE_2_ by 83% in rat gingiva. They also showed that *n*−3 FAs significantly reduced LTC_4_ production as compared to rats of corn oil fed control group [88]. Similarly, a higher dietary intake of DHA is also associated with a lower prevalence of periodontitis [89]. Some animal studies also report an increase in the concentration of the anti-inflammatory cytokine IL−10 by *n*−3 FAs [90]. In addition to cytokines, cell culture models and animal studies have demonstrated decreased expression of adhesion molecules on endothelial cells, macrophages and lymphocytes that were exposed to *n*−3 FAs [91,92]. High expression of adhesion molecules is associated with inflammation [93].

In animal models of periodontitis, *n*−3 FAs are found to be substrates for neutrophil production of resolvins and protectins, both key mediators in the resolution of inflammation [94,95]. A recent study reported lipid mediator profiles that differed between healthy, periodontitis and treated periodontitis in gingival tissue [96]. In periodontitis patients prior to treatment, increased levels of SPM pathway markers were detected due to the increased activity of SPM synthesis [96]. Lipoxin A_4_ had a high detection frequency in periodontitis patients prior to treatment compared to the after treatment and control groups. This finding reflects an increased activity of the LX pathway in periodontitis [97]. Conversely, the SPM pathway marker (for both leukotrienes and lipoxins) 5-HETE is also higher in periodontitis patients [96]. Another study found increased levels of 15-HETE and 5-HETE in saliva and whole blood samples in patients with aggressive periodontitis as compared to healthy controls. This shows elevated omega−6-driven pro-resolving as well as pro-inflammatory activities [98]. SPMs also enhance the release of fibroblast growth factor (FGF) from human periodontal ligament (PDL) cells, stimulate non-phlogistic macrophage recruitment and formation of pro-resolving macrophages, which are vital for tissue regeneration [99,100]. A recent study by Kantarci and colleagues demonstrated the expression of SPM receptors GPR32 and ALX/FPR2 in PDL fibroblasts. The application of RvD1 (100 nM) not only reversed IL−1β-induced inhibition of wound healing and proliferation of PDLF, but also the production of pro-inflammatory cytokines and matrix metalloproteinases [101].

### 4.3. Protective Functions in Bone Metabolism

Bone resorption is a result of an imbalance between the activity of bone forming osteoblasts and bone resorbing osteoclasts. Alveolar bone loss is one of the main characteristics of periodontitis which, if uncontrolled, eventually culminates in tooth loss. The receptor activator of nuclear factor kappa B (RANK) and its ligand (RANKL) are key in osteoclast proliferation and differentiation signaling. RANKL is expressed by many cells, including osteoblasts, fibroblasts and T cells and its production is regulated in response to the presence of inflammatory cytokines such as TNF-α and IL−1 [102,103,104].

**Figure 3:**
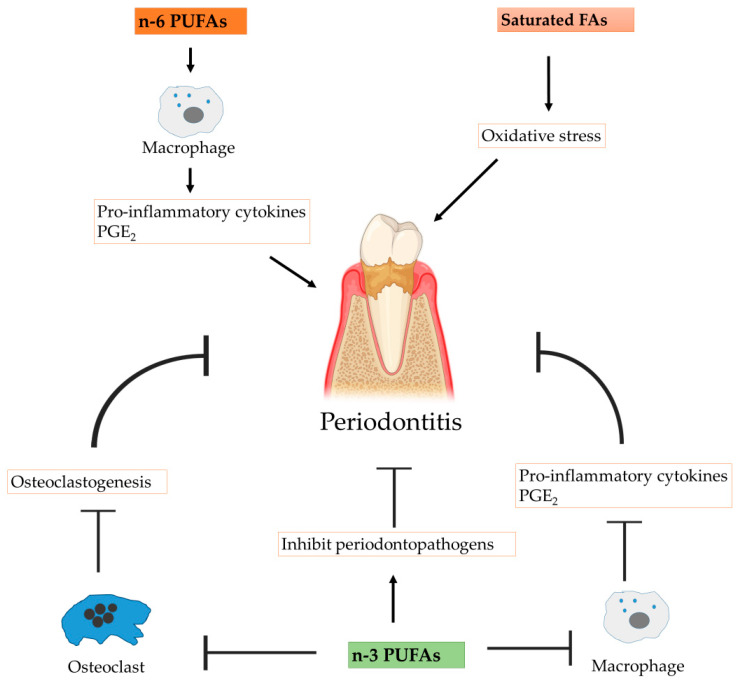
Implications of fatty acids in periodontitis.

*n*−3 FAs (both EPA and DHA) stimulate osteoblast survival by activating pro-survival Akt signaling and suppressing the glucocorticoid-induced pro-death pathway [105]. This is due to their anti-inflammatory actions which modulate PPAR-γ signaling and lower levels of inflammatory cytokines such as IL−1, IL−6 and TNF-α, whilst suppressing AA-derived synthesis of eicosanoids including PGE_2_ [106,107]. In addition to enhancing osteoblastic activity, both EPA and DHA have been shown to promote osteoblastogenesis and prevent bone resorption by altering membrane function and regulating calcium balance [108]. The role of *n*−3 FAs in modulating inflammatory bone loss is a positive one as they are inversely associated with periodontal alveolar bone loss (Figure 3) [109,110,111]. Their direct antimicrobial actions may influence periodontal pathogenesis by inhibiting putative periodontopathogens and reducing the strength of the biological stimulus [112]. Indirectly, *n*−3 FAs are anti-inflammatory and modulate the synthetic pathways for many inflammatory mediators including IL−1, IL−6 and TNF-α (as discussed above). Both DHA and AA can decrease bone resorption by suppressing the expression of osteoclast-specific genes including *NFATc1, CTSK, TRAP, c-Fos, MMP−9* and *DC-STAMP* in differentiating osteoclasts, thus reducing their overall numbers [113,114,115]. In addition, DHA and AA inhibit the migration and adhesion of osteoclasts by downregulating expression of RANK and vitronectin receptor (or VNR, which helps mediate the attachment of the cells to the bone matrix) [116]. The anti- osteoclastogenic strategy is further strengthened by DHA’s ability to trigger apoptosis of mature osteoclasts by inducing Bim expression, a Bcl−2 family protein [117].

The immunoresolving actions of SPMs are far more potent than their parent compounds. The first study showing bone-protective actions of LXs in addition pro-resolution was in a rabbit model of periodontal disease where topical treatment with 6 µg of the LX analog ASA-triggered LXA_4_ three times a week diminished alveolar bone loss [118]. The same study provided the first in vivo evidence for RvE1’s bone-protective actions. Periodontal disease was induced in New Zealand white rabbits via application of silk ligature and the periodontal pathogen *P. gingivalis* to the second mandibular premolar. RvE1 was topically administered (4 µg) three times per week. Evaluation after 6 weeks showed a significant inhibition of bone loss, determined by morphometric analysis and radiography [118]. The direct action of RvE1 on osteoclasts was determined by in vitro studies using murine bone marrow–derived primary osteoclasts. RvE1 was administered to primary osteoclast cultures in nanomolar doses (3–30 nM), which markedly decreased the number and size of differentiated osteoclasts induced by macrophage colony-stimulating factor and RANKL [119]. RvE1 can directly target BLT1 receptors on osteoclasts to inhibit osteoclast fusion and maturation, while inducing the release of osteoprotegerin (OPG) to antagonize the resorptive role of osteoclast-stimulating RANKL, and thus facilitates the prevention of alveolar bone loss [119]. Th17 cells are potent inducers of osteoclastogenesis. They do so by secreting IL−17, RANKL, TNF, IL−1, and IL−6 [120]. The secreted IL−17 then stimulates the release of RANKL by osteoblasts and therefore potentiates osteoclastogenic activity of RANKL by upregulating RANK [121]. RvE1, RvD1, RvD2 and MaR prevent IL−17 expression and IL−17A secretion by Th17 cells [122].

### 4.4. Antimicrobial Actions

In addition to their anti-inflammatory actions, *n*−3 FAs also exhibit antimicrobial activity. Both EPA and DHA inhibit the activity of periodontal pathogens, such as *Porphyromonas gingivalis, Fusobacterium nucleatum,* and *Prevotella intermedia* [112]. Huang and Ebersole reported on the strong antibacterial activity of both EPA and DHA against oral pathogens, including *Streptococcus mutans, Candida albicans,* and *Porphyromonas gingivalis* (at 50% inhibitory concentration from 1 to 10 μg/mL) [123].

Similar findings regarding EPA and DHA’s antimicrobial activity on mature biofilms grown on hydroxyapatite discs were reported recently where both EPA and DHA significantly reduced the bacterial counts and cell viability in an in vitro multispecies biofilm model (*Streptococcus oralis*, *Actinomyces naeslundii*, *Veillonella parvula*, *Fusobacterium nucleatum*, *Porphyromonas gingivalis*, and *Aggregatibacter actinomycetemcomitans*) [124]. The underlying mechanisms for *n*−3 FAs’ antibacterial effect are still unknown. It might be that the incorporation of EPA and DHA into the cell plasma membrane results in greater membrane fluidity and permeability, which in turn would compromise its integrity, eventually leading to cell death [125,126]. Interestingly, the presence of unsaturated double bonds can exert a toxic effect directly on the bacterial cell membrane [127].

## 5. Conclusions

There is a large body of evidence that clearly shows the positive modulating actions of *n*−3 FAs, especially EPA and DHA, in periodontitis. The intake of EPA and DHA is associated with reduced inflammation, bone loss and increased clinical attachment gain, all desirable endpoints in periodontal therapy. Their use as adjuncts may be of paramount relevance as host modulating agents in patients who respond poorly to conventional treatment. Future research should be aimed at capturing the potency of SPMs in resolving inflammation and making them a leading class of therapeutic agents in resolution pharmacology. Until that time, the use of dietary *n*−3 FAs will suffice in the prevention and halting of inflammation in the periodontal tissues.

## Data Availability

Not applicable.

## References

[1] Lamont R.J., Koo H., Hajishengallis G. (2018). The oral microbiota: Dynamic communities and host interactions. Nat. Rev. Microbiol..

[2] Serhan C.N., Chiang N., Van Dyke T.E. (2008). Resolving inflammation: Dual anti-inflammatory and pro-resolution lipid mediators. Nat. Rev. Immunol..

[3] Panezai J., Van Dyke T.E. (2022). Resolution of inflammation: Intervention strategies and future applications. Toxicol. Appl. Pharmacol..

[4] Wiktorowska-Owczarek A., Berezinska M., Nowak J.Z. (2015). PUFAs: Structures, metabolism and functions. Adv. Clin. Exp. Med..

[5] Pereira S.L., Leonard A.E., Mukerji P. (2003). Recent advances in the study of fatty acid desaturases from animals and lower eukaryotes. Prostaglandins Leukot Essent Fat. Acids.

[6] Burdge G.C. (2006). Metabolism of alpha-linolenic acid in humans. Prostaglandins Leukot Essent Fat. Acids.

[7] Abeywardena M.Y., Patten G.S. (2011). Role of ω3 long chain polyunsaturated fatty acids in reducing cardio-metabolic risk factors. Endocr. Metab. Immune Disord.

[8] Das U.N. (2006). Essential fatty acids—A review. Curr. Pharm. Biotechnol..

[9] Giltay E.J., Gooren L.J., Toorians A.W., Katan M.B., Zock P.L. (2004). Docosahexaenoic acid concentrations are higher in women than in men because of estrogenic effects. Am. J. Clin. Nutr..

[10] Khanapure S.P., Garvey D.S., Janero D.R., Gordon Letts L. (2007). Eicosanoids in inflammation: Biosynthesis, pharmacology, and therapeutic frontiers. Curr. Top. Med. Chem..

[11] von Moltke J., Trinidad N.J., Moayeri M., Kintzer A.F., Wang S.B., van Rooijen N., Brown C.R., Krantz B.A., Leppla S.H., Gronert K. (2012). Rapid Induction of Inflammatory Lipid Mediators by the Inflammasome in Vivo. Nature.

[12] Bozza P.T., Bakker-Abreu I., Navarro-Xavier R.A., Bandeira-Melo C. (2011). Lipid body function in eicosanoid synthesis: An update. Prostagl. Leukot.Essent. Fat. Acids.

[13] Vane J.R., Bakhle Y.S., Botting R.M. (1998). Cyclooxygenases 1 and 2. Annu. Rev. Pharmacol. Toxicol..

[14] Sergeant S., Rahbar E., Chilton F.H. (2016). Gamma-linolenic acid, dihommo-gamma linolenic, eicosanoids and inflammatory processes. Eur. J. Pharmacol..

[15] Serhan C.N., Petasis N.A. (2011). Resolvins and protectins in inflammation resolution. Chem. Rev..

[16] Kuhn H., Banthiya S., van Leyen K. (2015). Mammalian lipoxygenases and their biological relevance. Biochim. Biophys. Acta.

[17] Feinmark S.J., Cannon P.J. (1986). Endothelial cell leukotriene C4 synthesis results from intercellular transfer of leukotriene A4 synthesized polymorphonuclear leukocytes. J. Biol. Chem..

[18] Serhan C.N. (2014). Pro-Resolving Lipid Mediators are Leads for Resolution Physiology. Nature.

[19] Spiecker M., Liao J.K. (2015). Vascular protective effects of cytochrome p450 epoxygenase-derived eicosanoids. Arch. Biochem. Biophys..

[20] Levy B.D., Clish C.B., Schmidt B., Gronert K., Serhan C.N. (2001). Lipid Mediator Class Switching During Acute Inflammation: Signals in Resolution. Nat. Immunol..

[21] Mori T.A., Hodgson J.M. (2013). Fatty acids. Encyclopedia of Human Nutrition.

[22] Thies F., Nebe-von-Caron G., Powell J.R., Yaqoob P., Newsholme E.A., Calder P.C. (2001). Dietary supplementation with γ -linolenic acid or fish oil decreases T lymphocyte proliferation in healthy older humans. J. Nutr..

[23] Kelley D.S., Taylor P.C., Nelson G.J., Mackey B.E. (1998). Arachidonic acid supplementation enhances synthesis of eicosanoids without suppressing immune functions in young healthy men. Lipids.

[24] Kew S., Banerjee T., Minihane A.M., Finnegan Y.E., Williams C.M., Calder P.C. (2003). Relation between the fatty acid composition of peripheral blood mononuclear cells and measures of immune cell function in healthy, free-living subjects aged 25-72 y. Am. J. Clin. Nutr..

[25] Simopoulos A.P., DiNicolantonio J.J. (2016). The importance of a balanced omega-6 to omega-3 ratio in the prevention and management of obesity. Open Heart.

[26] Calder P.C. (2007). Immunomodulation by omega-3 fatty acids. Prostaglandins Leukot. Essent. Fat. Acids.

[27] DiNicolantonio J.J., O’Keefe J.H. (2018). Importance of maintaining a low omega-6/omega-3 ratio for reducing inflammation. Open Heart.

[28] Fujikawa M., Yamashita N., Yamazaki K., Sugiyama E., Suzuki H., Hamazaki T. (1992). Eicosapentaenoic Acid Inhibits Antigen-Presenting Cell Function of Murine Splenocytes. Immunology.

[29] Calder P.C., Bevan S.J., Newsholme E.A. (1992). The Inhibition of T-lymphocyte Proliferation by Fatty Acids Is via an Eicosanoid-Independent Mechanism. Immunology.

[30] Betz M., Fox B.S. (1991). Prostaglandin E2 Inhibits Production of Th1 Lymphokines but Not of Th2 Lymphokines. J. Immunol..

[31] Snijdewint F.G.M., Kalinski P., Wierenga E.A., Bos J.D., Kapsenberg M.L. (1993). Prostaglandin E2 Differentially Modulates Cytokine Secretion Profiles of Human T Helper Lymphocytes. J. Immunol..

[32] Kunkel S.L., Chensue S.W., Phan S.H. (1986). Prostaglandins as Endogenous Mediators of Interleukin-1 Production. J. Immunol..

[33] Renz H., Gong J.-H., Schmidt A., Nain M., Gemsa D. (1988). Release of Tumor Necrosis Factor-α from Macrophages: Enhancement and Suppression Are Dose-Dependently Regulated by Prostaglandin E2 and Cyclic Nucleotides. J. Immunol..

[34] Rode H.N., Szamel M., Schneider S., Resch K. (1982). Phospholipid Metabolism of Stimulated Lymphocytes. Preferential Incorporation of Polyunsaturated Fatty Acids into Plasma Membrane Phospholipid upon Stimulation with Concanavalin A. Biochim. Biophys. Acta.

[35] Sreeramkumar V., Fresno M., Cuesta N. (2012). Prostaglandin E2 and T cells: Friends or foes?. Immunol. Cell Biol..

[36] Raman P., Kaplan B.L., Kaminski N.E. (2013). 15-Deoxy-Delta(1)(2),(1)(4)- prostaglandin J(2)-glycerol, a putative metabolite of 2-arachidonoyl glycerol and a peroxisome proliferator-activated receptor gamma ligand, modulates nuclear factor of activated T cells. J. Pharmacol. Exp. Ther..

[37] Kumar G.S., Das U.N. (1994). Effect of prostaglandins and their precursors on the proliferation of human lymphocytes and their secretion of tumor necrosis factor and various interleukins. Prostaglandins Leukot Essent Fat. Acids.

[38] Xue L., Barrow A., Fleming V.M., Hunter M.G., Ogg G., Klenerman P., Pettipher R. (2011). Leukotriene E4 activates human Th2 cells for exaggerated proinflammatory cytokine production in response to prostaglandin D2. J. Immunol..

[39] Ariel A., Chiang N., Arita M., Petasis N.A., Serhan C.N. (2003). Aspirin-Triggered Lipoxin A 4 and B 4 Analogs Block Extracellular Signal-Regulated Kinase-Dependent TNF-α Secretion from Human T Cells. J. Immunol..

[40] Harbige L.S. (2003). Fatty acids, the immune response, and autoimmunity: A question of n− 6 essentiality and the balance between n− 6 and n− 3. Lipids.

[41] Matono R., Miyano K., Kiyohara T., Sumimoto H. (2014). Arachidonic acid induces direct interaction of the p67(phox)-Rac complex with the phagocyte oxidase Nox2, leading to superoxide production. J. Biol. Chem..

[42] Pompeia C., Cury-Boaventura M.F., Curi R. (2003). Arachidonic acid triggers an oxidative burst in leukocytes. Braz. J. Med. Biol. Res..

[43] Norris P.C., Dennis E.A. (2012). Omega-3 fatty acids cause dramatic changes in TLR4 and purinergic eicosanoid signaling. Proc. Natl. Acad. Sci. USA.

[44] Zhang Y., Chen H., Zhang W., Cai Y., Shan P., Wu D., Zhang B., Liu H., Khan Z.A., Liang G. (2020). Arachidonic acid inhibits inflammatory responses by binding to myeloid differentiation factor-2 (MD2) and preventing MD2/toll-like receptor 4 signaling activation. Biochim. Et Biophys. Acta (BBA)-Mol. Basis Dis..

[45] Marion-Letellier R., Butler M., Dechelotte P., Playford R.J., Ghosh S. (2008). Comparison of cytokine modulation by natural peroxisome proliferator-activated receptor gamma ligands with synthetic ligands in intestinal-like Caco-2 cells and human dendritic cells—Potential for dietary modulation of peroxisome proliferator-activated receptor gamma in intestinal inflammation. Am. J. Clin. Nutr..

[46] Yaqoob P., Pala H.S., Cortina-Borja M., Newsholme E.A., Calder P.C. (2000). Encapsulated fish oil enriched in -tocopherol alters plasma phospholipid and mononuclear cell fatty acid compositions but not mononuclear cell functions. Eur. J. Clin. Investig..

[47] Healy D.A., Wallace F.A., Miles E.A., Calder P.C., Newsholme P. (2000). The effect of low to moderate amounts of dietary fish oil on neutrophil lipid composition and function. Lipids.

[48] Yaqoob P., Calder P.C. (1995). Effects of dietary lipid manipulation upon inflammatory mediator production by murine macrophages. Cell Immunol..

[49] Lee T.H., Mencia-Huerta J.M., Shih C., Corey E.J., Lewis R.A., Austen K.F. (1984). Characterization and biologic properties of 5,12-dihydroxy derivatives of eicosapentaenoic acid, including leukotriene-B5 and the double lipoxygenase product. J. Biol. Chem..

[50] Bagga D., Wang L., Farias-Eisner R., Glaspy J.A., Reddy S.T. (2003). Differential effects of prostaglandin derived from w-6 and w-3 polyunsaturated fatty acids on COX-2 expression and IL-6 secretion. Proc. Natl. Acad. Sci. USA.

[51] Wada M., DeLong C.J., Hong Y.H., Rieke C.J., Song I., Sidhu R.S., Yuan C., Warnock M., Schmaier A.H., Yokoyama C. (2007). Enzymes and receptors of prostaglandin pathways with arachidonic acid-derived versus eicosapentaenoic acid-derived substrates and products. J. Biol. Chem..

[52] Gorjao R., Verlengia R., de Lima T.M., Soriano F.G., Boaventura M.F.C., Kanunfre C.C., Peres C.M., Sampaio S.C., Otton R., Folador A. (2006). Effect of docosahexaenoic acid-rich fish oil supplementation on human leukocyte function. Clin. Nutr..

[53] Miles E.A., Banerjee T., Dooper M.M., M'Rabet L., Graus Y.M., Calder P.C. (2004). The influence of different combinations of gamma-linolenic acid, stearidonic acid and EPA on immune function in healthy young male subjects. Br. J. Nutr..

[54] Siga L.H. (2006). Basic science for the clinician 39: NF-kappaB-function, activation, control, and consequences. J. Clin. Rheumatol..

[55] Perkins N.D. (2007). Integrating cell-signalling pathways with NF-kappaB and IKK function. Nat. Rev. Mol. Cell Biol..

[56] Babcock T.A., Novak T., Ong E., Jho D.H., Helton W.S., Espat N.J. (2002). Modulation of lipopolysaccharide-stimulated macrophage tumor necrosis factor-a production by w-3 fatty acid is associated with differential cyclooxygenase-2 protein expression and is independent of interleukin-10. J. Surg. Res..

[57] Novak T.E., Babcock T.A., Jho D.H., Helton W.S., Espat N.J. (2003). NF-kappa B inhibition by omega -3 fatty acids modulates LPS-stimulated macrophage TNF-alpha transcription. Am. J. Physiol..

[58] Carlsson J.A., Wold A.E., Sandberg A.S., Ostman S.M. (2015). The Polyunsaturated Fatty Acids Arachidonic Acid and Docosahexaenoic Acid Induce Mouse Dendritic Cells Maturation but Reduce T-Cell Responses in Vitro. PLoS ONE.

[59] Kim W., Fan Y.Y., Barhoumi R., Smith R., McMurray D.N., Chapkin R.S. (2008). n-3 polyunsaturated fatty acids suppress the localization and activation of signaling proteins at the immunological synapse in murine CD4+ T cells by affecting lipid raft formation. J. Immunol..

[60] Hou T.Y., Barhoumi R., Fan Y.Y., Rivera G.M., Hannoush R.N., McMurray D.N., Chapkin R.S. (2016). n-3 polyunsaturated fatty acids suppress CD4(+) T cell proliferation by altering phosphatidylinositol-(4,5) -bisphosphate [PI(4,5)P-2] organization. Biochim. Et Biophys. Acta-Biomembr..

[61] Fan Y.Y., Fuentes N.R., Hou T.Y., Barhoumi R., Li X.C., Deutz N.E.P., Engelen M.P.K.J., McMurray D.N., Chapkin R.S. (2018). Remodelling of primary human CD4(+) T cell plasma membrane order by n-3 PUFA. Br. J. Nutr..

[62] Zech T., Ejsing C.S., Gaus K., de Wet B., Shevchenko A., Simons K., Harder T. (2009). Accumulation of raft lipids in T-cell plasma membrane domains engaged in TCR signalling. EMBO J..

[63] Cucchi D., Camacho-Munoz D., Certo M., Niven J., Smith J., Nicolaou A., Mauro C. (2020). Omega-3 polyunsaturated fatty acids impinge on CD4+ T cell motility and adipose tissue distribution via direct and lipid mediator-dependent effects. Cardiovasc. Res..

[64] Onodera T., Fukuhara A., Shin J., Hayakawa T., Otsuki M., Shimomura I. (2017). Eicosapentaenoic acid and 5-HEPE enhance macrophage-mediated Treg induction in mice. Sci. Rep..

[65] Serhan C.N., Dalli J., Colas R.A., Winkler J.W., Chiang N. (2015). Protectins and maresins: New pro-resolving families of mediators in acute inflammation and resolution bioactive metabolome. Biochim. Biophys Acta Mol Cell Biol Lipids.

[66] Serhan C.N., Dalli J., Karamnov S., Choi A., Park C.K., Xu Z.Z., Ji R.R., Zhu M., Petasis N.A. (2012). Macrophage proresolving mediator maresin 1 stimulates tissue regeneration and controls pain. FASEB J..

[67] Dalli J., Zhu M., Vlasenko N.A., Deng B., Haeggström J.Z., Petasis N.A., Serhan C.N. (2013). The novel 13S,14S-epoxy-maresin is converted by human macrophages to maresin 1 (MaR1), inhibits leukotriene A4 hydrolase (LTA4H), and shifts macrophage phenotype. FASEB J..

[68] Matte A., Recchiuti A., Federti E., Koehl B., Mintz T., El Nemer W., Tharaux P.L., Brousse V., Andolfo I., Lamolinara A. (2019). Resolution of sickle cell disease associated inflammation and tissue damage with 17R-resolvin D1. Blood J. Am. Soc. Hematol..

[69] Norling L.V., Spite M., Yang R., Flower R.J., Perretti M., Serhan C.N. (2011). Cutting edge: Humanized nano-proresolving medicines mimic inflammation-resolution and enhance wound healing. J. Immunol..

[70] Eke P.I., Dye B.A., Wei L., Thornton-Evans G.O., Genco R.J. (2012). Prevalence of periodontitis in adults in the United States: 2009 and 2010. J. Dent. Res..

[71] Kassebaum N.J., Smith A.G., Bernabé E., Fleming T.D., Reynolds A.E., Vos T., Murray C.J., Marcenes W., GBD 2015 Oral Health Collaborators (2017). Global, regional, and national prevalence, incidence, and disability-adjusted life years for oral conditions for 195 countries, 1990–2015: A systematic analysis for the global burden of diseases, injuries, and risk factors. J. Dent. Res..

[72] Kosaka T., Ono T., Yoshimuta Y., Kida M., Kikui M., Nokubi T., Maeda Y., Kokubo Y., Watanabe M., Miyamoto Y. (2014). The effect of periodontal status and occlusal support on masticatory performance: The Suita study. J. Clin. Periodontol..

[73] Genco R.J., Borgnakke W.S. (2013). Risk factors for periodontal disease. Periodontol 2000.

[74] Hajishengallis G. (2014). The inflammophilic character of the periodontitis-associated microbiota. Mol. Oral. Microbiol..

[75] Tonetti M.S., Greenwell H., Kornman K.S. (2018). Staging and grading of periodontitis: Framework and proposal of a new classification and case definition. J. Clin. Periodontol..

[76] Loos B.G., Van Dyke T.E. (2020). The role of inflammation and genetics in periodontal disease. Periodontol. 2000.

[77] Ballini A., Cantore S., Dedola A., Santacroce L., Laino L., Cicciù M., Mastrangelo F. (2018). IL-1 haplotype analysis in periodontal disease. J. Biol. Regul. Homeost. Agents.

[78] Scarel-Caminaga R.M., Kim Y.J., Viana A.C., Curtis K.M., Corbi S.C., Sogumo P.M., Orrico S.R., Cirelli J.A. (2011). Haplotypes in the interleukin 8 gene and their association with chronic periodontitis susceptibility. Biochem. Genet..

[79] Nibali L., Pelekos G., D’Aiuto F., Chaudhary N., Habeeb R., Ready D., Parkar M., Donos N. (2013). Influence of IL-6 haplotypes on clinical and inflammatory response in aggressive periodontitis. Clin. Oral Investig..

[80] Anovazzi G., Medeiros M.C., Pigossi S.C., Finoti L.S., Mayer M.P., Rossa C., Scarel-Caminaga R.M. (2017). Functional haplotypes in interleukin 4 gene associated with periodontitis. PLoS ONE.

[81] Magnusson I., Lindhe J., Yoneyama T., Liljenberg B. (1984). Recolonization of a subgingival microbiota following scaling in deep pockets. J. Clin. Periodontol..

[82] Cortellini P., Buti J., Pini Prato G., Tonetti M.S. (2017). Periodontal regeneration compared with access flap surgery in human intra-bony defects 20-year follow-up of a randomized clinical trial: Tooth retention, periodontitis recurrence and costs. J. Clin. Periodontol..

[83] Preshaw P.M. (2008). Host response modulation in periodontics. Periodontol. 2000.

[84] Kruse A.B., Kowalski C.D., Leuthold S., Vach K., Ratka-Krüger P., Woelber J.P. (2020). What is the impact of the adjunctive use of omega-3 fatty acids in the treatment of periodontitis? A systematic review and meta-analysis. Lipids Health Dis..

[85] Araghizadeh N., Paknejad M., Alaeddini M., Minaii B., Abdollahi M., Khorasanie R. (2014). The efficacy and prophylactic characteristics of omega-3 fatty acids in experimental gingivitis in rats. Iran J. Basic Med. Sci..

[86] Hankenson K.D., Watkins B.A., Schoenlein I.A., Allen K.G., Turek J.J. (2000). Omega-3 fatty acids enhance ligament fibroblast collagen formation in association with changes in interleukin-6 production. Proc. Soc. Exp. Biol. Med..

[87] Caughey G.E., Mantzioris E., Gibson R.A., Cleland L.G., James M.J. (1996). The effect on human tumor necrosis factor α and interleukin 1β production of diets enriched in n-3 fatty acids from vegetable oil or fish oil. Am. J. Clin. Nutr..

[88] Alam S.Q., Bergens B.M., Alam B.S. (1991). Arachidonic acid, prostaglandin E2 and leukotriene C4 levels in gingiva and submandibular salivary glands of rats fed diets containing n-3 fatty acids. Lipids.

[89] Naqvi A.Z., Buettner C., Phillips R.S., Davis R.B., Mukamal K.J. (2010). n-3 fatty acids and periodontitis in US adults. J. Am. Diet. Assoc..

[90] Sierra S., Lara-Villoslada F., Comalada M., Olivares M., Xaus J. (2008). Dietary eicosapentaenoic acid and docosahexaenoic acid equally incorporate as decosahexaenoic acid but differ in inflammatory effects. Nutrition.

[91] Collie-Duguid E., Wahle K. (1996). Inhibitory effect of fish oil n−3 polyunsaturated fatty acids on the expression of endothelial cell adhesion molecules. Biochem. Biophys. Res. Commun..

[92] Hughes D., Southon S., Pinder A. (1996). (n−3) Polyunsaturated fatty acids modulate the expression of functionally associated molecules on human monocytes in vitro. J. Nutr..

[93] Albelda S., Smith C., Ward P. (1994). Adhesion molecules and inflammatory injury. FASEB J..

[94] Serhan C.N., Savill J. (2005). Resolution of inflammation: The beginning programs the end. Nat. Immunol..

[95] Levy B.D., Kohli P., Gotlinger K., Haworth O., Hong S., Kazani S., Israel E., Haley K.J., Serhan C.N. (2007). Protectin D1 is generated in asthma and dampens airway inflammation and hyperresponsiveness. J. Immunol..

[96] Ferguson B., Bokka N.R., Maddipati K.R., Ayilavarapu S., Weltman R., Zhu L., Chen W., Zheng W.J., Angelov N., Van Dyke T.E. (2020). Distinct profiles of specialized pro-resolving lipid mediators and corresponding receptor gene expression in periodontal inflammation. Front. Immunol..

[97] Pouliot M., Clish C.B., Petasis N.A., Van Dyke T.E., Serhan C.N. (2000). Lipoxin A(4) analogues inhibit leukocyte recruitment to Porphyromonas gingivalis: A role for cyclooxygenase-2 and lipoxins in periodontal disease. Biochemistry.

[98] Elabdeen H.R., Mustafa M., Szklenar M., Ruhl R., Ali R., Bolstad A.I. (2013). Ratio of pro-resolving and pro-inflammatory lipid mediator precursors as potential markers for aggressive periodontitis. PLoS ONE.

[99] Hong S., Porter T.F., Lu Y., Oh S.F., Pillai P.S., Serhan C.N. (2008). Resolvin E1 metabolome in local inactivation during inflammation-resolution. J. Immunol..

[100] Dalli J., Serhan C.N. (2012). Specific lipid mediator signatures of human phagocytes: Microparticles stimulate macrophage efferocytosis and pro-resolving mediators. Blood J. Am. Soc. Hematol..

[101] Zarrough A.E., Hasturk H., Stephens D.N., Van Dyke T.E., Kantarci A. (2022). Resolvin D1 modulates periodontal ligament fibroblast function. J. Periodontol..

[102] Boyle W.J., Simonet W.S., Lacey D.L. (2003). Osteoclast differentiation and activation. Nature.

[103] Hofbauer L.C., Heufelder A.E. (2001). Role of receptor activator of nuclear factor-kappaB ligand and osteoprotegerin in bone cell biology. J. Mol. Med..

[104] Nakashima T., Kobayashi Y., Yamasaki S., Kawakami A., Eguchi K., Sasaki H., Sakai H. (2000). Protein expression and functional difference of membrane-bound and soluble receptor activator of NFkappaB ligand: Modulation of the expression by osteotropic factors and cytokines. Biochem. Biophys. Res. Commun..

[105] Candelario J., Tavakoli H., Chachisvilis M. (2012). PTH1 receptor is involved in mediating cellular response to long-chain polyunsaturated fatty acids. PLoS ONE.

[106] Yeh L.C.C., Ford J.J., Lee J.C., Adamo M.L. (2014). Palmitate attenuates osteoblast differentiation of fetal rat calvarial cells. Biochem. Biophys. Res Commun..

[107] Kruger M.C., Coetzee M., Haag M., Weiler H. (2010). Long-chain polyunsaturated fatty acids: Selected mechanisms of action on bone. Prog. Lipid Res..

[108] Kruger M.C., Coetzee M., Haag M., Weiler H. (2013). Long-chain polyunsaturated fatty acids may mutually benefit both obesity and osteoporosis. Nutr. Res..

[109] Azuma M.M., Gomes-Filho J.E., Cardoso C.D.B.M., Pipa C.B., Narciso L.G., Bomfim S.R.M., Jacinto R.D.C., Cintra L.T.A. (2018). Omega 3 fatty acids reduce the triglyceride levels in rats with apical periodontitis. Braz. Dent. J..

[110] Bendyk A., Marino V., Zilm P.S., Howe P., Bartold P.M. (2009). Effect of dietary omega-3 polyunsaturated fatty acids on experimental periodontitis in the mouse. J. Periodontal. Res..

[111] Li Y., Lu Z., Zhang X., Yu H., Kirkwood K.L., Lopes-Virella M.F., Huang Y. (2015). Metabolic syndrome exacerbates inflammation and bone loss in periodontitis. J. Dent. Res..

[112] Choi J.S., Park N.H., Hwang S.Y., Sohn J.H., Kwak I., Cho K.K., Choi I.S. (2013). The antibacterial activity of various saturated and unsaturated fatty acids against several oral pathogens. J. Environ. Biol..

[113] Kasonga A.E., Deepak V., Kruger M.C., Coetzee M. (2015). Arachidonic acid and do- cosahexaenoic acid suppress osteoclast formation and activity in human CD14+ monocytes, in vitro. PLoS ONE.

[114] Rahman M.M., Bhattacharya A., Fernandes G. (2008). Docosahexaenoic acid is more potent inhibitor of osteoclast differentiation in RAW 264.7 cells than eicosapentaenoic acid. J. Cell Physiol..

[115] Boeyens J.C., Deepak V., Chua W.H., Kruger M.C., Joubert A.M., Coetzee M. (2014). Effects of omega 3-and omega 6-polyunsaturated fatty acids on RANKL-induced osteoclast differentiation of RAW264.7 cells: A comparative in vitro study. Nutrients.

[116] Nakamura I., Duong L.T., Rodan S.B., Rodan G.A. (2007). Involvement of alpha(v) beta3 integrins in osteoclast function. J. Bone Miner. Metab..

[117] Kim H.J., Ohk B., Yoon H.J., Kang W.Y., Seong S.J., Kim S.Y., Yoon Y.R. (2017). Docosahexaenoic acid signaling attenuates the proliferation and differentiation of bone marrow-de- rived osteoclast precursors and promotes apoptosis in mature osteoclasts. Cell Signal.

[118] Hasturk H., Kantarci A., Ohira T., Arita M., Ebrahimi N., Chiang N., Petasis N.A., Levy B.D., Serhan C.N., Van Dyke T.E. (2006). RvE1 protects from local inflammation and osteoclast- mediated bone destruction in periodontitis. FASEB J..

[119] Herrera B.S., Ohira T., Gao L., Omori K., Yang R., Zhu M., Muscara M.N., Serhan C.N., Van Dyke T.E., Gyurko R. (2008). An endogenous regulator of inflammation, resolvin E1, modulates osteoclast differentiation and bone resorption. Br. J. Pharmacol..

[120] Jovanovic D.V., Di Battista J.A., Martel-Pelletier J., Jolicoeur F.C., He Y., Zhang M., Mineau F., Pelletier J.P. (1998). IL-17 stimulates the production and expression of proinflammatory cytokines, IL-beta and TNF-alpha, by human macrophages. J. Immunol..

[121] Pacifici R. (2016). The role of IL-17 and TH17 cells in the bone catabolic activity of PTH. Front. Immunol..

[122] Chiurchiù V., Leuti A., Dalli J., Jacobsson A., Battistini L., Maccarrone M., Serhan C.N. (2016). Pro-resolving lipid mediators Resolvin D1, Resolvin D2 and Maresin 1 are critical in modulating T cell responses. Sci. Transl. Med..

[123] Huang C.B., Ebersole J.L. (2010). A novel bioactivity of omega-3 polyunsaturated fatty acids and their ester derivatives. Mol. Oral Microbiol..

[124] Ribeiro-Vidal H., Sánchez M.C., Alonso-Español A., Figuero E., Ciudad M.J., Collado L., Herrera D., Sanz M. (2020). Antimicrobial activity of EPA and DHA against oral pathogenic bacteria using an in vitro multi-species subgingival biofilm model. Nutrients.

[125] Desbois A.P., Smith V.J. (2010). Antibacterial free fatty acids: Activities, mechanisms of action and biotechnological potential. Appl. Microbiol. Biotechnol..

[126] Desbois A.P. (2012). Potential applications of antimicrobial fatty acids in medicine, agriculture and other industries. Recent Pat. Antiinfect Drug Discov..

[127] Correia M., Michel V., Matos A.A., Carvalho P., Oliveira M.J., Ferreira R.M., Dillies M.A., Huerre M., Seruca R., Figueiredo C. (2012). Docosahexaenoic acid inhibits Helicobacter pylori growth in vitro and mice gastric mucosa colonization. PLoS ONE.

